# Immunity as a predictor of anti-malarial treatment failure: a systematic review

**DOI:** 10.1186/s12936-017-1815-y

**Published:** 2017-04-20

**Authors:** Katherine O’Flaherty, Julia Maguire, Julie A. Simpson, Freya J. I. Fowkes

**Affiliations:** 1 Macfarlane Burnet Institute of Medical Research, Melbourne, VIC 3004 Australia; 2 Centre for Epidemiology and Biostatistics, Melbourne School of Population and Global Health, The University of Melbourne, Melbourne, 3010 Australia; 3 Department of Epidemiology and Preventive Medicine and Department of Infectious Diseases, Monash University, Melbourne, 3800 Australia

**Keywords:** Malaria, Immunity, Antibodies, Antimalarials, Treatment efficacy, Treatment failure, Systematic review

## Abstract

**Background:**

Naturally acquired immunity can reduce parasitaemia and potentially influence anti-malarial treatment outcomes; however, evidence for this in the current literature provides conflicted results. The available evidence was synthesized to determine and quantify the association between host immunity and anti-malarial treatment failure.

**Methods:**

Four databases were searched to identify studies investigating malaria antibody levels in patients receiving anti-malarial treatment for symptomatic malaria with treatment failure recorded according to the World Health Organization classification. Odds ratios or hazard ratios were extracted or calculated to quantify the association between malarial antibody levels and treatment failure, and findings from different studies were visualized using forest plots.

**Results:**

Eight studies, including patients with falciparum malaria treated with mono- and combination therapy of artemisinin derivatives, sulfadoxine, pyrimethamine and chloroquine, were identified. Reported and calculated effect estimates varied greatly between studies, even those assessing the same antigens and treatments. An association between blood-stage IgG responses and treatment efficacy was observed. The greatest magnitudes of effect were observed for artemisinin [OR/HR (95% CI) range 0.02 (0.00, 0.45)–1.08 (0.57, 2.06)] and chloroquine [0.24 (0.04, 1.37)–0.32 (0.05, 1.96)] treatments, and larger magnitudes of effect were observed for variant surface antigen responses [0.02 (0.00, 0.45)–1.92 (0.94, 3.91)] when compared with merozoite specific responses [0.24 (0.04, 1.37)–2.83 (1.13, 7.09)].

**Conclusions:**

Naturally acquired malarial immunity is associated with reduced anti-malarial treatment failure in malaria endemic populations. Anti-malarial IgG effects treatment outcome differently for different anti-malarial drugs and antigen targets, and had the greatest impact during treatment with the current first-line treatments, the artemisinins. This has implications for the assessment of the therapeutic efficacy of anti-malarials, particularly in the context of emerging artemisinin resistance.

**Electronic supplementary material:**

The online version of this article (doi:10.1186/s12936-017-1815-y) contains supplementary material, which is available to authorized users.

## Background

Malaria is a major public health problem, an estimated 215 million clinical cases and more than 400,000 malaria-related deaths occurred in 2015 alone [1]. The World Health Organization (WHO) currently recommends artemisinin-based combination therapy (ACT) as the first-line treatment for all falciparum malaria [2]. Worryingly, the efficacy of the artemisinins is declining due to the emergence of slow-clearing *Plasmodium falciparum* parasites after artemisinin treatment in patients throughout Southeast Asia [3, 4]. Widespread treatment failure of artemisinin derivatives is yet to be reported but previous first-line anti-malarial treatments, such as chloroquine and sulfadoxine-pyrimethamine have been phased out due to drug resistance and treatment failure [5, 6].

Anti-malarial treatment outcome is determined, according to WHO criteria, as either adequate clinical and parasitological response (ACPR) or treatment failure, which can be further categorized as early treatment failure (ETF), late clinical failure (LCF), or late parasitological failure (LPF) [7, 8]. The predominant cause of treatment failure is resistance to the active drug, or in the case of combination therapy, resistance to one or more of the active components. However, the efficacy of anti-malarials may be influenced by other factors independent of the parasites susceptibility to the drugs. For example, patients vary greatly in their drug concentration versus time profiles, the parasite burden and age distribution of the parasites at initial treatment, and the level of within-host immunity to malaria [9].

Naturally acquired immunity to malaria develops in an age-dependant manner, after repeated exposure, in individuals living in malaria-endemic regions (reviewed in [10, 11]). Antibodies targeting the blood stage of *Plasmodium* spp. are acquired with age and are an important component of the anti-malarial immune response, acting by reducing parasite density and clinical symptoms [12, 13]. Treatment efficacy improves with increasing age and intensified transmission, suggesting that acquired immunity may play a role in determining the efficacy of anti-malarial treatments [14–17]. The direct role that naturally acquired immunity plays in influencing anti-malarial treatment outcome has been investigated in several studies with conflicting conclusions. The aim of this systematic review was to synthesize the evidence of studies investigating the relationship between *Plasmodium*-specific blood-stage antibody responses and anti-malarial treatment failure. In addition, variations in the association according to the anti-malarial administered (which have different pharmacokinetic-pharmacodynamic profiles) and blood-stage antibody response (which can target different antigens and parasite life-cycle stages) was investigated.

## Methods

A systematic review of the literature was performed according to PRISMA (preferred reporting items for systematic reviews and meta-analyses) (see Prisma Checklist, Additional file 1) [18] and MOOSE (meta-analysis of observational studies in epidemiology) guidelines [19].

### Search methods for the identification of studies

Databases Pubmed, Scopus, Web of Science and LILACS (Latin American and Caribbean Health Sciences Literature) were searched independently by two review authors (KO and JM) for studies examining the association between malarial immunity and anti-malarial treatment outcomes for all years up to and including 9 January, 2017 (Additional file 2). Keywords included: malaria, immunity, treatment, *Plasmodium*, *P. falciparum*, *Plasmodium viva*x, anti-malarial, antibody, IgG, chloroquine, quinine, amodiaquine, proguanil, sulfadoxine, pyrimethamine, mefloquine, artemisinin, dihydroartemisinin, artesunate, atovaquone, artemether, lumefantrine, piperaquine, apical membrane antigen, erythrocyte binding antigen, merozoite surface protein, and glutamate rich protein. The search included articles published in all languages. The abstracts of returned articles were assessed for potential relevance and full articles were retrieved. Reference lists of studies identified through database searches were also investigated to identify additional studies for this review.

### Criteria for considering studies

#### Study designs

Cohort studies including randomized and non-randomized controlled efficacy trials of anti-malarial drugs and nested case–control studies were included. Cross-sectional studies and mathematical models were excluded.

#### Study participants

Individuals, including pregnant women, living in a malaria-endemic region and receiving treatment for uncomplicated or severe malaria caused by any *Plasmodium* spp. were included.

#### Antibody measures

Total immunoglobulin G (IgG) responses to *Plasmodium* spp. parasites and infected erythrocytes (IEs), as well as recombinant and synthetic representatives of blood-stage antigens, were included. Studies investigating proxies of blood-stage immunity such as age, transmission intensity or antibodies specific for sporozoite and gametocyte antigens were excluded.

#### Treatment failure measures

The revised WHO Classification of treatment failures (ACPR, ETF, LCF, LPF) was used to define treatment outcome and is summarized in Table 1 [7]. Results were limited to this WHO measure of treatment failure to ensure maximum comparability between studies; other measures of treatment response, such as parasite clearance, varied greatly between studies (e.g., parasite clearance time, parasite clearance half-life, parasite reduction ratio at 48 h, etc.), and were excluded from analyses.

**Table 1:** Description of malaria treatment outcomes

Malaria treatment outcome	Description
Revised WHO classification of treatment failures [7, 8]^a^	Early treatment failure (ETF)
	Development of danger signs or severe malaria on days 1, 2 or 3, in the presence of parasitaemia
	Parasitaemia on day 2 higher than day 0 count, irrespective of axillary temperature
	Parasitaemia on day 3 with axillary temperature of ≥37.5 °C
	Parasitaemia of day 3 ≥25% of day 0 count
	Late treatment failure
	Late clinical failure (LCF)
	Development of danger signs or severe malaria after day 3 in presence of parasitaemia
	Presence of parasitaemia and axillary temperature ≥37.5 °C on any day from day 4 to 14 (day 28 in low transmission areas), without previously meeting any of the criteria of ETF
	Late parasitological failure (LPF)
	Presence of parasitaemia on day 14 (any day from day 7 to 28 in low transmission areas), and axillary temperature ≥37.5 °C, without previously meeting any of the criteria of ETF or LCF
	Adequate clinical and parasitological response (ACPR)
	Absence of parasitaemia on day 28 irrespective of axillary temperature without previously meeting any of the criteria of ETF or LCF or LPF

^a^Separate protocols followed in low and high transmission areas until 2002, after this one protocol is implemented and recommends the systematic use of PCR as well as a new 28 or 42 day follow up period. Applicable to all anti-malarial treatments

#### Quality criteria

The minimum quality criterion was parasitaemia confirmed by light microscopy or PCR. Treatment outcome was considered for single *Plasmodium* spp. infections only, therefore mixed infections were excluded. Two authors independently assessed each article against inclusion and quality criteria and extracted descriptive information, with discrepancies resolved by discussion with all authors.

### Data collection

Measures of association (odds ratio (ORs), hazards ratio (HRs), mean differences and median differences) as well as 95% confidence intervals (CIs), standard errors (SE), standard deviations (SD) and the proportion of treatment failures and successes were extracted or calculated independently by two authors. Adjusted estimates were reported where possible. If an OR/HR was equal to 1, participants seropositive for antibodies were seen as having the same odds (hazard) of treatment failure as participants that were seronegative. A relative difference of 25% in odds or hazard of treatment failure between antibody seropositive and seronegative groups was defined a priori and considered clinically meaningful. Where ORs or HRs could not be extracted or calculated, mean differences in antibody levels between treatment failures and successes were calculated with 95% CIs, these studies were referred to in narrative terms only. On the occasion that two publications utilized the same patient data, only the study with the largest sample size was included. Studies that assessed treatment failures following combination therapy were grouped according to the most potent parasiticidal drug (artemisinin derivatives in all instances [20]). If a study measured antibody levels at multiple time points (days 0 (baseline), 7, 14, and 28 being the most common measurement points), only antibody measurements taken at day 0 (i.e., prior to administration of anti-malarial treatment), were included. Authors of the original articles were contacted and asked to provide estimates or data which could be used to generate estimates if key data were omitted from the published study. No retrospective changes were made to the protocol while performing the review, with a risk of bias assessment was added during the peer review process and all included studies underwent an individual risk of bias assessment using the risk of bias in non-randomized studies-of interventions tool (ACROBAT-NRSI) [21].

## Results

### Identification and characteristics of included studies

**Fig. 1:**
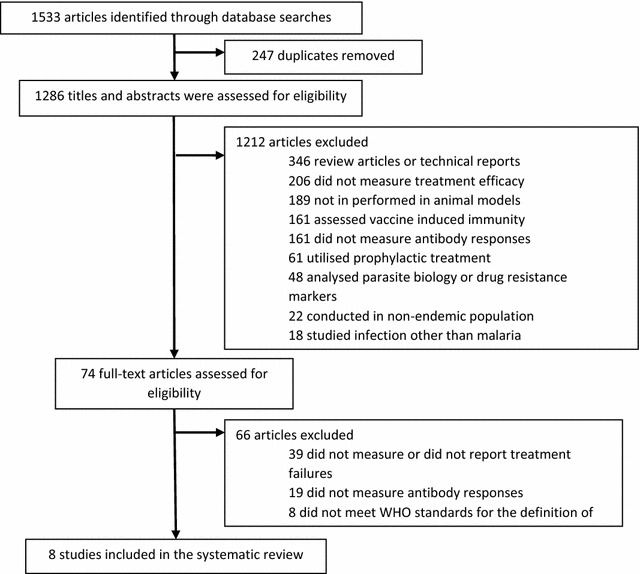
Flow chart of study identification. The characteristics of identified studies are provided in Table 2

Database searches identified 1286 articles (1533 less 247 duplicate articles) with 74 full text articles assessed for eligibility and a final eight studies fulfilling the inclusion and quality criteria (study identification detailed in Fig. 1). Included studies underwent individual risk of bias assessment, with all studies classified as having a low-moderate risk of bias (Additional file 3). The main criteria for moderate risk of bias being no adjustment for potential confounding. All eight included studies examined patients infected with *P. falciparum,* and included clinical efficacy cohort studies, randomized control trials and nested case–control studies. Participants were recruited with uncomplicated malaria in seven studies [22–28], one study included participants with uncomplicated and severe malaria [29], and two excluded patients with severe malaria from participation [25, 28]. All studies included active follow-up of patients until at least day 28 (Table 2). The majority of the studies (n = 7) were conducted in Africa in children under 15 years old [22–28], with one conducted in Southeast Asia that reported only adult participants [29] (Table 2). First-line artemisinin derivatives, artemether (AM) and artesunate (AS), were assessed in two studies as either monotherapy (AM [28, 29], AS [29]), in combination with lumefantrine (LM) [28, 29] or with the antibiotic azithromycin (AZ) [29]. The most common anti-malarial treatments studied were amodiaquine (AQ) and sulfadoxine-pyrimethamine (SP), which were assessed as mono- or combination therapy in five of the eight studies [22–25, 27]. A further two studies investigated the association between *P. falciparum* antibody responses and chloroquine treatment failure (CQ) [22, 26] (Table 2). The included studies reported total IgG responses to antigens *P. falciparum* merozoite antigens (MSP1 [23, 27], MSP1-19 [22, 24–26], MSP1 Block 2 proteins [23], MSP2 [24, 28], MSP3 [22, 24], EBA-175 [27], AMA-1 [24, 26–28]) and antigens expressed on the surface of *Pf*-IE (VSAs [27, 28], RESA [29],), as well as schizont extract [27]. Two studies reported IgG responses to the glutamate rich protein (GLURP) [22, 24], which is expressed in multiple parasite life stages but was analysed with merozoite specific responses (Table 2).

**Table 2:** Characteristics of studies included in the systematic review

Study: author, year	Country (province)	Study design (n)	Age range (years)	Antigen (allele)—IgG seroprevalence	Antimalarial	Dosage	Follow-up (days)	Treatment failure (n/N)
Van Geertruyden, 2009 [26]	Zambia (Copperbelt)	Randomized control trial (268)	15–50	AMA1^b^, MSP2_(3D7)_, MSP2_(FC27)_, VSA_(E8B)_, VSA_(A4)_, VSA_(HCD6)_^f^	AM + LM or SP^f^	*AM* + *LM*: 20 mg AM and 120 mg LM at 0, 8, 25, 36, 48 and 60 h; *SP:* 500 mg S and 25 mg P × 3 tablets as a single dose	45	11% (30/268)^h^
Mayxay, 2001 [27]	Thailand (Central Region)	Clinical efficacy study (80)	24 (mean)^a^	RESA^b^—80%	AS or AS + AZ or AM + LM^f^	DNS	28	50% (40/80)^h^
Enevold, 2007 [25]	Tanzania (Dodoma Region)	Clinical efficacy study (100)	0.5–<5	AMA1_(FVO)_—75%, DBL2βPF13_0003_(3D7)_—85%, DBL4PFD1235W_(3D7)_—76%, EBA-175^b^—62%, MSP1^b^—85%, MSP3_(FVO)_—82%, CIDR1_(3D7)_—72%, GLURP-R0_(FVO)_—70%, GLURP-R2_(FVO)_—65%, VSA1^c^—82%, VSA2^d^ —82%, Schizont Extract _(F32)_—95%	SP (n = 50) or AQ (n = 50)	*SP;* 25 mg/kg S and 1.25 mg/kg P once daily for 3 days; *AQ:* 10 mg/kg AQ once daily for 3 days	28	DNS
Keh, 2012 [22]	Uganda (Central Region)	Clinical efficacy study (88)	1–10	AMA1_(3D7)_—63%, MSP1-19_(FVO)_—95%, MSP2_(3D7)_—87%, MSP3^b^—15%, GLURPR0_(F32)_—26%, GLURPR2_(F32)_—40%	AQ + SP (n = 88)	*AQ* + *SP:* 25 mg/kg AQ over 3 days (10, 10, 5 mg/kg) and 25 mg/kg S and 1.25 mg/kg P on day 1	63	11% (10/88)
Mawili-Mboumba, 2003 [21]	Gabon (Moyen-Ogooué)	Clinical efficacy study (153)	0.5–10	MSP1Bl2_(K1)_—43%, MSP1 Bl2_(RO33)_—16%, MSP1 Bl2_(MAD20)_—10%, MSP1 Bl1—83%	AQ (n = 153)	*AQ:* 10 mg/kg per day for 3 days	28	33% (51/153)
Aubuoy, 2007 [23]	Gabon (Hauut-Ogooué)	Clinical efficacy study (232)	0.5–10	MSP1-19_(Wellcome)_^e^	AQ (n = 118) or SP (n = 114)	*AQ:* 30 mg/kg AQ days 0 and 1; *SP:* 25 mg/kg S and 1.25 mg/kg P days 0 and 1	28	AQ: 32% (38/118) SP: 14% (16/114)
Diarra, 2012 [20]	Burkina-Faso (Bazega)	Clinical efficacy study (284)	0.5–15	MSP1-19^b^, MSP3^b^, GLURP^b,e^	CQ (n = 195) or SP (n = 53)	*CQ:* 25 mg/kg CQ over 3 days (10, 10, 5 mg/kg); *SP:* 500 mg S and 25 mg P^g^	28	CQ: 62% (33/53) SP: 92% (179/195)
Pinder, 2006 [24]	The Gambia (Kerewan)	Clinical efficacy study (46)	1–10	AMA1_(FVO)_—76%, MSP1-19_(Wellcome)_—80%	CQ (n = 46)	*CQ:* 25 mg/kg over 3 days (10, 10, 5 mg/kg)	28	36% (17/46)

*SP* sulphadoxine–pyrimethamine, *AQ* amodiaquine, *DHA* dihydroartemisinin, *AS* artesunate, *AM* artemether, *LM* lumefantrine, *AZ* Azithromycin, *CQ* chloroquine, *DNS* did not state

^a^Range of ages not provided

^b^Allele not stated by authors

^c^3D7 unselected VSA

^d^3D7 selected VSA on transformed human bone marrow endothelial cells

^e^seroprevalence data not shown

^f^Patients not stratified by treatment given

^g^Dosages not provided, taken from the 2003 WHO guidelines as stated by the paper

^h^Treatment failure not stratified by treatment arm

### Associations between antibody responses to *Plasmodium falciparum* blood stages and artemisinin-based mono- or combination therapy treatment failure

**Fig. 2:**
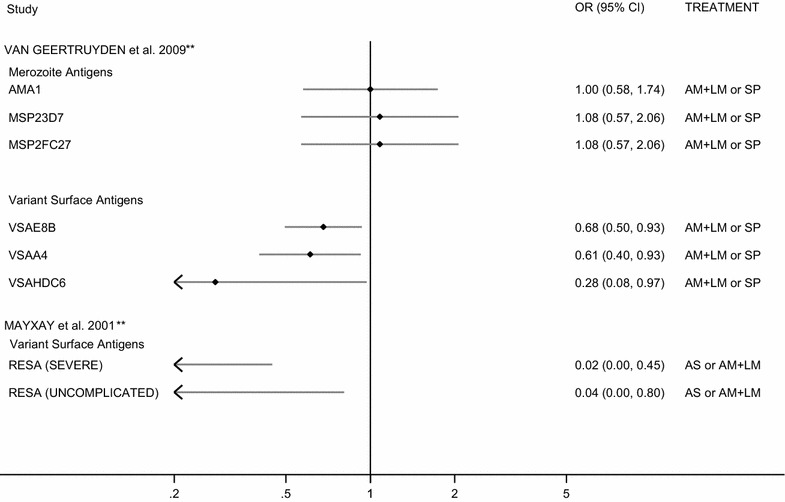
Forest plot of the association between IgG seroprevalence and ACT treatment failure. ORs correspond to the odds of treatment failure after treatment with artemisinin containing mono- or combination therapy for seropositive versus seronegative individuals. *Estimates are calculated by the authors from data in the published paper, **estimates are published estimates. All estimates are unadjusted with the exception of Van Geertruyden et al. [28], which are adjusted for CD4+ count on day 0. Estimates for patients stratified by disease severity are reported where applicable. *OR* odds ratio, *AM* artemether, *LM* lumefantrine, *SP* sulfadoxine pyrimethamine, *AS* artesunate

Two studies examined AM-LM efficacy [28, 29], one of which also examined treatment failure in patients administered AS monotherapy [29]. Van Geertruyden et al. in Zambia showed no association between the presence of high levels of anti-merozoite IgG and odds of LPF with AM-LM (OR, AMA1 = 1 (95% CI 0.58,1.75); MSP2_3D7_ = 1.08 (0.57, 2.07); MSP2_FC27_ = 1.08 (0.57, 2.07) (Fig. 2; Table 2) but demonstrated a reduced odds of LPF by 32–72% in patients seropositive for anti-VSA antibodies specific for individual strains [OR, E8B = 0.68 (0.50, 0.94); A4 = 0.61 (0.40, 0.92); HCD6 = 0.28 (0.08, 0.96)] (Fig. 2; Table 2) in patients with uncomplicated malaria [28]. In concordance with these observations, Mayxay et al. in Thailand demonstrated a large reduction in the odds of LPF and LCF with AS monotherapy ± AZ or AM-LM by 96 and 98% in patients positive for anti-RESA IgG with uncomplicated and severe malaria, respectively [OR 0.04 (0.00, 0.80) 0.02 (0.00, 0.45), respectively] [29] (Fig. 2; Table 2).

### Associations between antibody responses to *Plasmodium falciparum* blood stages and amodiaquine or sulfadoxine-pyrimethamine mono- or combination therapy treatment failure

**Fig. 3:**
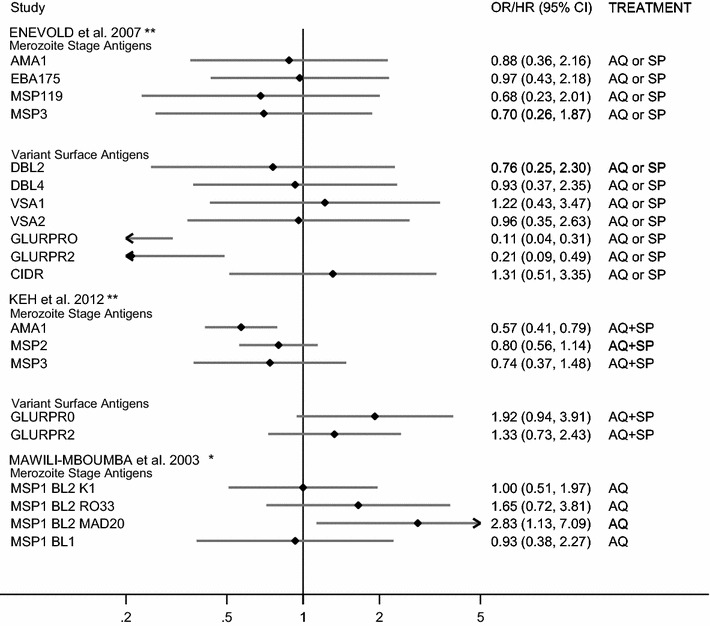
Forest plot of the association between IgG antibody responses and AQ/SP treatment failure. ORs correspond to the odds of treatment failure after treatment with artemisinin containing mono- or combination therapy for seropositive versus seronegative individuals. *Estimates are calculated by the authors from data in the published paper, **estimates are published estimates. All estimates are unadjusted with the exception of Keh et al. [24], which are adjusted for age and parasite polymorphism. All estimates are displayed as ORs, with the exception of the estimates from Keh et al. which are displayed as hazard ratios (HR). *AQ* amodiaquine, *SP* sulfadoxine pyrimethamine

Four studies investigated the association between blood-stage antibodies and AQ or SP monotherapy [22, 23, 25, 27] and one investigated AQ-SP combination therapy [24] in patients with uncomplicated malaria. The relationship between IgG specific for different *P. falciparum* blood-stage targets and treatment efficacy differed greatly both within and between studies. Enevold et al. and Keh et al. determined IgG levels to a broad panel of merozoite and *Pf*-IE antigens, but only a small number of responses were found to be associated with reduced odds of treatment failure (4/11 and 2/5, respectively, Fig. 3) [24, 27]. Enevold and colleagues demonstrated that Tanzanian patients positive for anti-GLURP_R2_ and anti-GLURP_R0_ IgG had a 79 and 89% reduction in odds of treatment failure, respectively, when treated with either an AQ or SP monotherapy [OR, GLURP_R2_ = 0.21 (0.09, 0.49) GLURP_R0_ = 0.11 (0.04, 0.31)], but no associations were found with merozoite responses or other VSA (Fig. 3) [27]. Keh and colleagues reported that in Ugandan patients ten-fold increases in anti-AMA1 IgG responses were associated with a 43% reduction in the risk of treatment failure when treated with an AQ-SP combination therapy [HR = 0.57 (0.41, 0.79)] and this trend was seen with the other merozoite antigens (Fig. 3) [24]. Conversely to Enevold et al., Keh et al. observed a trend towards increased odds of treatment failure in those with anti-GLURP antibodies [OR, GLURP_R0_ = 1.92 (0.94, 3.89); GLURP_R2_ = 1.33 (0.73, 2.44)] (Fig. 3) [24]. Similarly, in a study in Gabon, Mawili-Mboumba et al. reported that the presence of IgG antibodies to MSP1_BL2_ antigens was associated with increased odds of AQ monotherapy treatment failure [OR, RO33 = 1.65 (0.72, 3.83); MAD20 = 2.83 (1.13, 7.10); K1 = 1.00 (0.51, 1.97)], but found MSP1_BL1_ specific IgG to associated with a very slight decrease in the odds of treatment failure [OR 0.93 (0.38, 2.27)] (Fig. 3) [23].

Where ORs or HRs could not be calculated, mean differences in antibody levels between treatment outcome groups were assessed. Overall, there was no association found between anti-merozoite or anti-*Pf*-IE specific antibodies and treatment outcome for patients receiving SP monotherapy, and anti-merozoite IgG were only slightly higher in the ACPR group compared to the treatment failure groups in a study by Diarra et al. in Burkina Faso (MSP1-19 mean difference = 0.11 (95% CI 0.18, 0.40), MSP-3 mean difference = 0.16 (−0.15, 0.47), GLURP mean difference IgG 0.11 (0.22, 0.44) [22]). Similarly, Anti-MSP1-19 IgG levels were marginally lower in the LCF and LPF groups when compared to the ACPR group in a study in Gabon conducted by Aubouy et al. [LCF group mean difference = 9.3 (−3.16, 21.76), LPF mean difference = 4.3 (−6.51, 15.11)], yet greater mean difference in antibody levels was observed between the ETF and ACPR groups in the same study (ETF group mean difference IgG = 48 (27, 69) [25].

### Associations between antibody responses to *Plasmodium falciparum* blood stages and chloroquine monotherapy treatment failure

**Fig. 4:**
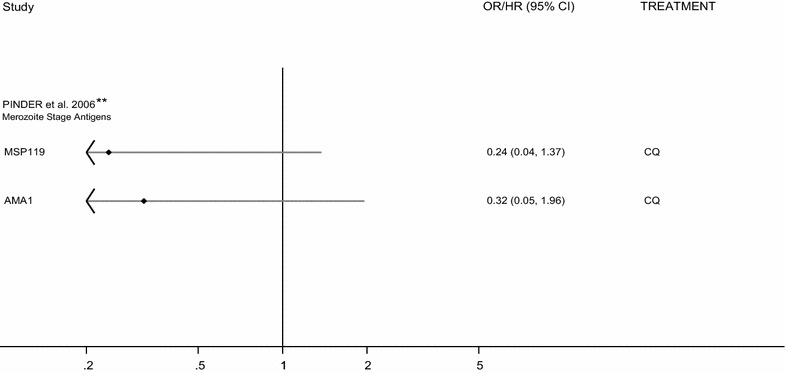
Forest plot of the association between IgG seroprevalence and CQ treatment failure. ORs correspond to the odds of treatment failure after treatment with artemisinin containing mono- or combination therapy for seropositive versus seronegative individuals. *Estimates are calculated by the authors from data in the published paper, **estimates are published estimates. All estimates are unadjusted with the exception of Pinder et al. [26], which are adjusted for age. *OR* odds ratio, *CQ* chloroquine

Two studies analysed treatment efficacy in patients administered CQ monotherapy [22, 26]. Pinder et al. described a 68 and 76% reduction in the odds of LCF in individuals seropositive for MSP1-19 and AMA1IgG, respectively, in Gambian children with uncomplicated malaria (OR 0.24 (95% CI 0.04, 1.37), 0.32 (0.05, 1.96), respectively, Fig. 4) [26]. Diarra et al. observed marginally lower levels of merozoite and *Pf*-IE specific antibodies [IgG, IgG subclasses and IgM) in patients receiving CQ monotherapy and experiencing TF, compared to those in the ACPR group (mean difference in total IgG specific for MSP1-19 0.06 (95% CI 0.35, 0.47) MSP3 = 0.37 (0.12, 0.62); GLURP = 0.43 (−0.02, 0.88)] [22].

## Discussion

Identifying and quantifying host factors that determine anti-malarial treatment efficacy is essential for monitoring the occurrence of treatment failures and emerging resistance. An association between antibodies specific for *P. falciparum* blood-stage antigens and treatment failure was found for each of the anti-malarials investigated, with the largest magnitude of effect observed for artemisinin derivatives and chloroquine. Heterogeneity was observed in these associations according to the blood-stage antigen under investigation, with larger magnitudes of effect observed for variant surface antigens compared to merozoite antigens.

The anti-malarial treatments included in this review have different pharmacokinetic and pharmacodynamic profiles. The largest magnitude of effect with blood-stage immunity and anti-malarial treatment efficacy was observed with artemisinin derivatives and chloroquine. The 4-aminoquinolines such as chloroquine, amodiaquine and piperaquine, as well as the artemisinins, target early parasite forms [20], particularly the ring stage in the case of artemisinins [30]. The combined targeting of early intra-erythrocytic parasites by antibodies and drugs which preferentially target early forms is likely to provide swift clearance of IEs before cyto-adhesion and sequestration can occur (>18 h post merozoite invasion) [31]. Conversely, treatment with dihydrofolate reductase inhibitors, such as pyrimethamine, interrupts late parasite stages (after the first 24 h of parasite life cycle) [20], leaving parasites to mature, and *Pf*-IEs to rosette and sequester regardless of treatment until the next parasite cycle. In addition to the variety of parasitic targets, the anti-malarials assessed in the included studies have different drug concentration–time profiles. The artemisinins, for instance, have very short elimination half-lives (between 0.7 and 1.4 h in the case of artesunate) (reviewed in [32]), when compared with chloroquine which has a long terminal elimination phase, and may be detected in the patient months after administration [33]. The difference observed in the drug elimination time profiles between treatments indicates that follow-up times recommended for determining treatments failures should vary in order to avoid underestimating treatment failure in those drugs with longer plasma half-lives [34], however only two studies observed patients beyond 28 days despite a myriad of treatments used across included studies [24, 28]. Immunity may therefore have a differential effect on the treatment failure of different anti-malarials, although this cannot definitively be concluded by this review as no individual study compared effects across different anti-malarials.

In most cases anti-*Pf*-IE antibodies were associated with the largest decrease in odds/risk of treatment failure compared to anti-merozoite antibodies, which suggests that immune mechanisms which contribute to *Pf*-IE clearance (e.g., opsonic phagocytosis) rather than by reducing parasite multiplication rates [35–38] may have a greater impact on measures of treatment failure. The varying magnitude of effect observed within merozoite antigens may also support a direct role of anti-merozoite responses in treatment failure. Antibodies specific for AMA-1, EBA-175 and MSPs antibodies, were found to reduce the odds of treatment failure [24, 26, 27] and have been associated with protection from high density parasitaemia and symptomatic malaria in other studies [12, 39], whereas anti-MSP1 Block 2 specific antibodies, were not associated with a reduced odds of treatment failure [23] and in previous studies have not been shown to be protective against high density parasitaemia and symptomatic infections [12]. Given that the antigen/parasite strain under investigation are potential sources of heterogeneity, both different antigens within study sites, and the same antigens across study sites (e.g., AMA1 and GLURP), further investigation into the relative utility of different antigens in assessing immunity in drug efficacy studies is warranted.

Any host mechanism capable of contributing to parasite clearance will have a profound effect in patients treated with drugs that are no longer or only partially efficacious by contributing to parasite clearance which may be wrongly interpreted as a direct effect of treatment. The frequency of drug-resistant parasites and malaria transmission. may also influence the association between antibodies and treatment failure. Pinder et al. [26] and Enevold et al. [27] examined the impact of immunity in a population where drug resistance was established but only one confirmed the presence of known molecular markers [26]. Furthermore, the presence of resistant parasites may further influence results, as it has been recently demonstrated that the largest effect of immunity on parasite clearance after artemisinin treatment was observed in patients harbouring artemisinin resistant *kelch13* mutant rather than wild-type parasites [40].. Differences in transmission intensity and acquisition of naturally acquired immunity between study sites may also be a source of heterogeneity. The majority of the included studies were conducted in moderate-high transmission settings [22–28], with only one study assessing treatment efficacy in a low-transmission setting in Thailand [29]. Despite being in an area of low transmission, and presumably of low naturally acquired immunity, this study by Mayxay et al. showed the highest magnitude of effect on the association between *Pf*-IE antibodies and reduced odds of artemisinin treatment failure. Findings in this systematic review may be generalizable to populations of varying transmission but the generalizability of findings to areas of varying frequencies of genetic mutations are yet to be determined.

A strength of this review was that studies published in all languages were included and authors were contacted to provide estimates and data for inclusion in the review. A further strength is that the WHO classification of anti-malarial treatment failures was utilized to ensure the inclusion of rigorous studies and maximum comparability between studies. Importantly, the current WHO guidelines for the assessment of antimalarial treatment efficacy requires the use of molecular genotyping in regions of intense transmission to ensure recrudescent infections are accurately recorded and ensure reinfection if not mistaken for treatment failure and for inclusion in treatment failure analyses {WHO, 2009 #2599}. Two of the included studies (both of which utilised data acquired prior to the recommendation of PCR correction in 2003 [7]) either did not complete or did not report molecular genotyping [22, 29], the consequence being that treatment failures may have been overestimated in these studies. Furthermore, not all of the included studies categorized patients into the treatment failure sub-categories: ETF, LCF and LPF. This made the direct comparison of studies challenging, but also prevented analyses stratified for the different stages of treatment failure. Some studies did not include effect estimates stratified by the treatment given. For example Van Geertruyden et al. and Mayxay et al. provided estimates for combined patients treated by different drugs or the same drugs in mono- and combination therapy [28, 29], making it difficult to determine the effect of antibody responses to treatment efficacy of specific anti-malarial regimens. Furthermore, analysis was stratified according to the potency of included treatments. However, the importance of partner drugs should not be underestimated in providing efficacious treatment, specifically in the ACTS where partner drugs provide essential and most importantly long-lasting anti-parasitic activity in combination with the more potent but short lived artemisinin derivatives.

Methodological heterogeneity meant that meta-analyses could not be performed and pooled estimates were unable to be calculated to quantify the overall effect of immunity on treatment efficacy, or assess publication bias. Furthermore, formal investigations of the presence of drug resistance markers and endemicity and other established cofactors influencing treatment success such as pharmacokinetic exposure, host genetics, and parasitaemia could not be assessed (and also rely on all included studies determining these parameters). Importantly, no study investigated the effect of acquired immunity on treatment outcomes for *P. vivax* infection, which is the most widely distributed *Plasmodium* species and is responsible for a significant proportion of the clinical burden of malaria in Southeast Asia [41]. Future studies addressing the association between immunity and treatment of non-*falciparum* cases are warranted.

This systematic review provides evidence that naturally acquired antibodies to blood-stage malaria are associated with reduced treatment failure to anti-malarials with different pharmacokinetic-pharmacodynamic properties. Immunity is therefore an important confounder in the assessment of treatment failures and emerging anti-malarial drug resistance in malaria endemic populations.

## Additional files

**Additional file 1.** Preferred Reporting Items for Systematic Reviews and Meta-Analyses (PRISMA) Statement.

**Additional file 2: Table S1.** Database Search Results. **S2.** Detailed Database Search Terms.

**Additional file 3.** Risk of Bias Assessment for Included Studies.

## Abbreviations

ACPR: adequate clinical and parasitological response
ACT: artemisinin combination therapy
ADCC: antibody dependant cellular cytotoxicity
AM: artemether
AMA-1: apical membrane antigen 1
AQ: amodiaquine
AS: artesunate
CI: confidence interval
CQ: chloroquine
EBA: erythrocyte binding antigen
ETF: early treatment failure
GLURP: glutamate rich protein
HR: hazards ratio
IE: infected erythrocyte
Ig: immunoglobulin
LCF: late clinical failure
LM: lumefantrine
LPF: late parasitological failure
MSP: merozoite surface protein
OR: odds ratio
PPQ: piperaquine
RESA: ring infected erythrocyte stage antigen
SD: standard deviation
SE: standard error
SP: sulfadoxine-pyrimethamine
TF: treatment failure
VSA: variant surface antigen
