# Supplementary material for: Immunity as a predictor of anti-malarial treatment failure: a systematic review
Source: Malar J. 2017 Apr 20;16:158. doi: 10.1186/s12936-017-1815-y (PMC5397737; doi:10.1186/s12936-017-1815-y)
Supplement: Supplementary file 2 — Additional file 2: Table S1. Database Search Results. S2. Detailed Database Search Terms. [file 12936_2017_1815_MOESM2_ESM.docx]

| S1: Database Search Results | | | |
| --- | --- | --- | --- |
| Database | **Search string** | **Qualifiers/Filters** | **Results** |
| Pubmed | malaria and immunity and treatment and (plasmodium or plasmodium falciparum or plasmodium vivax) and (antimalarial or antibody or IgG or chloroquine or quinine or amodiaquine or proguanil or sulphadoxine or sulfadoxine or pyrimethamine or mefloquine or artemisinin or dihydroartemisinin or artesunate or atovaquone or artemether or lumefantrine or piperaquine or apical membrane antigen or erythrocyte binding antigen or merozoite surface protein or glutamate rich protein) | Species: Human  Publication date to 09/01/2017 | 822 |

| **S2: Detailed Search** | |
| --- | --- |
| immunity | "immunity"[MeSH Terms] OR "immunity"[All Fields] |
| malaria | "malaria"[MeSH Terms] OR "malaria"[All Fields] |
| treatment | "therapy"[Subheading] OR "therapy"[All Fields] OR "treatment"[All Fields] OR "therapeutics"[MeSH Terms] OR "therapeutics"[All Fields] |
| plasmodium | "plasmodium"[MeSH Terms] OR "plasmodium"[All Fields] |
| plasmodium falciparum | "plasmodium falciparum"[MeSH Terms] OR ("plasmodium"[All Fields] AND "falciparum"[All Fields]) OR "plasmodium falciparum"[All Fields] |
| plasmodium vivax | "plasmodium vivax"[MeSH Terms] OR ("plasmodium"[All Fields] AND "vivax"[All Fields]) OR "plasmodium vivax"[All Fields] |
| antimalarial | "antimalarials"[Pharmacological Action] OR "antimalarials"[MeSH Terms] OR "antimalarials"[All Fields] OR "antimalarial"[All Fields] |
| antibody | "immunoglobulins"[MeSH Terms] OR "immunoglobulins"[All Fields] OR "antibody"[All Fields] OR "antibodies"[MeSH Terms] OR "antibodies"[All Fields] |
| chloroquine | "chloroquine"[MeSH Terms] OR "chloroquine"[All Fields] |
| quinine | "quinine"[MeSH Terms] OR "quinine"[All Fields] OR "cinchona"[MeSH Terms] OR "cinchona"[All Fields] |
| amodiaquine | "amodiaquine"[MeSH Terms] OR "amodiaquine"[All Fields] |
| proguanil | "proguanil"[MeSH Terms] OR "proguanil"[All Fields] |
| sulphadoxine | "sulfadoxine"[MeSH Terms] OR "sulfadoxine"[All Fields] OR "sulphadoxine"[All Fields] |
| sulfadoxine | "sulfadoxine"[MeSH Terms] OR "sulfadoxine"[All Fields] |
| pyrimethamine | "pyrimethamine"[MeSH Terms] OR "pyrimethamine"[All Fields] |
| mefloquine | "mefloquine"[MeSH Terms] OR "mefloquine"[All Fields] |
| artemisinin | "artemisinine"[Supplementary Concept] OR "artemisinine"[All Fields] OR "artemisinin"[All Fields] OR "artemisinins"[MeSH Terms] OR "artemisinins"[All Fields] |
| dihydroartemisinin | "dihydroartemisinin"[Supplementary Concept] OR "dihydroartemisinin"[All Fields] |
| artesunate | "artesunate"[Supplementary Concept] OR "artesunate"[All Fields] |
| atovaquone | "atovaquone"[MeSH Terms] OR "atovaquone"[All Fields] |
| artemether | "artemether"[Supplementary Concept] OR "artemether"[All Fields] |
| lumefantrine | "lumefantrine"[Supplementary Concept] OR "lumefantrine"[All Fields] |
| piperaquine | "piperaquine"[Supplementary Concept] OR "piperaquine"[All Fields] |
| antigen | "antigens"[MeSH Terms] OR "antigens"[All Fields] OR "antigen"[All Fields] |
| membrane | "membranes"[MeSH Terms] OR "membranes"[All Fields] OR "membrane"[All Fields] |
| erythrocyte | "erythrocytes"[MeSH Terms] OR "erythrocytes"[All Fields] OR "erythrocyte"[All Fields] |
| merozoite | "merozoites"[MeSH Terms] OR "merozoites"[All Fields] OR "merozoite"[All Fields] |
| surface protein | "membrane proteins"[MeSH Terms] OR ("membrane"[All Fields] AND "proteins"[All Fields]) OR "membrane proteins"[All Fields] OR ("surface"[All Fields] AND "protein"[All Fields]) OR "surface protein"[All Fields] |
| glutamate | "glutamic acid"[MeSH Terms] OR ("glutamic"[All Fields] AND "acid"[All Fields]) OR "glutamic acid"[All Fields] OR "glutamate"[All Fields] OR "glutamates"[MeSH Terms] OR "glutamates"[All Fields] |
| protein | "proteins"[MeSH Terms] OR "proteins"[All Fields] OR "protein"[All Fields] |
