# Supplementary material for: Immunity as a predictor of anti-malarial treatment failure: a systematic review
Source: Malar J. 2017 Apr 20;16:158. doi: 10.1186/s12936-017-1815-y (PMC5397737; doi:10.1186/s12936-017-1815-y)
Supplement: Supplementary file 3 — Additional file 3. Risk of Bias Assessment for Included Studies. [file 12936_2017_1815_MOESM3_ESM.docx]

| Additional file 3: Risk of bias in individual studies | |
| --- | --- |
| Study: Author, Year | **Risk*** |
| Van Geertruyden, 2009 | Moderate |
| Mayxay, 2001 | Moderate |
| Enevold, 2007 | Low-moderate |
| Keh, 2012 | Moderate |
| Mawili-Mboumba, 2003 | Low-moderate |
| Aubuoy, 2007 | Moderate |
| Diarra, 2012 | Low-moderate |
| Pinder, 2006 | Low |
| *Assessed using the Risk Of Bias in Non-randomized Studies – of Interventions Tool | |
